# An Essay on Curvatures and Diseases of the Spine, Including All the Forms of Spinal Distortion

**Published:** 1845-03

**Authors:** 


Art. V.—*An Essay on Curvatures and Diseases of the Spine, including all the forms of Spinal Distortion:* to which the Fothergillian gold medal was awarded by the Medical Society of London. By R. W. Bampfield, Esq., one of the Surgeons to the Royal Metropolitan Infirmary for the Diseases of Children; Fellow of the Medical Society of London; Author of an Essay on Hemeralopia, or Night Blindness, of Practical Treatises on Tropical and Scorbutic Dysentery, &c. Edited by J. K. Mitchell, M. D., Professor of the Practice of Medicine in Jefferson Medical College of Philadelphia, &c., &c. Philadelphia: Ed. Barrington & Geo. D. Haswell. 1845. 12mo. pp. 223.

This volume makes the number of the *Select Medical Library* for October, and forms a seasonable contribution to our medical literature. The profession in the west is much in need of a work devoted to this specialty. Diseases of the spine are of not infrequent occurrence amongst us, and as the habits of our people become more luxurious they are likely to increase. It is therefore a matter of great importance that physicians should have their attention directed to the subject, and should be familiar with the earliest indications of the disease, in order that they may be able to meet it at its commencement, when, alone, it is treated with any certainty of success. Much suffering, and much hopeless deformity might be prevented if parents and practitioners were sufficiently on their guard against the first encroachments of these terrible maladies. "Whenever," is the just remark of the editor in his brief preface, "a child complains of unwonted lassitude, of tenderness in the back, or of constant pain in the side when seated, or drags its legs awkwardly, or begins to trip and fall in an inexplicable manner; or still more, when the dressmaker finds it difficult to obtain a 'neat fit,' or the shoulder-blade projects, or the line of the shoulders is inclined, we should examine well the spine, both as to sensibility and shape."

*Excurvation* of the spine is the disease first spoken of, and is considered by Mr. Bampfield the most frequent of all the distortions. Dr. Mitchell agrees that it is so in boys, who are seldom affected with any other form, but he maintains that the lateral curvature is much more frequent in girls. The treatment of it resolves itself into two classes — prevention and cure. The first consists in placing the patient in the recumbent posture, which is to be done at the earliest period.

"Generally speaking, in all cases of incipient curvature, the recumbent posture should be much adopted, with such a degree of exercise of the dorsal muscles as each particular case will admit of; but if inflammation be present, exercise may be injurious; if rheumatism exist, it cannot be employed, and if the remote cause be sprain, contusion, &c., the pain from attempting exercise will prevent its use."

"Some of the indications in the treatment of curvature of the spine are general, and will apply to its several varieties, whilst others will be particular, and peculiarly adapted to one variety only. The general indications applicable to all, are, to afford to the vertebral column effectual relief from pressure; to secure the bodies of the affected vertebræ from the irritation of motion in the erect attitude; to assist or enable the vertebral column to regain its true spinal line; to remove all constitutional and local symptoms of disease, and rectify all disordered functions, to promote the general health."

"The first indication will be fulfilled by relieving and protecting the spine from *all* superincumbent or other weight."

"The means adopted for the purpose of taking off all pressure from the spinal pyramid, have been to confine the body in the recumbent posture, either on an horizontal or an inclined plane; and it should be determined, on due consideration, if one of these contrivances be preferable to the other, or if either of them be objectionable."

"The second indication will be fulfilled by rest in the horizontal posture, and by abstaining from all movements and positions whilst so *lying*, which throw weight on the bodies of the vertebræ. This seems necessary, because movements of the body in the erect attitude would, sometimes, produce a degree of friction and motion of the diseased vertebræ that would be injurious; because they would tend to excite inflammation and absorption; and because analogy points it out, as absolute rest is enjoined in almost all diseases of other joints."

*Incurvation* of the spine is less frequent than the foregoing species. It is rarely attended, moreover, with the serious consequences that ensue from the other curvatures, and takes place generally, if not always, when the result of disease, in the cervical, lumbar, or lower dorsal vertebræ. The lumbar incurvation is generally removable at will; the cervical is permanent. The treatment, as in the preceding, consists mainly in directing the horizontal position. If the incurvation be of the cervical vertebræ, "the chin should be drawn in towards the neck; if of the loins, the flexion of the thighs tends to remove it." But this flexion cannot be constantly employed, because it would bring about permanent contraction in the flexor muscles of the thighs.

"Proper position will also contribute to the recovery, by taking off the superincumbent weight and pressure of the head, and by assisting in keeping the cervical vertebræ extended. For these purposes, the patient should have his head fixed in a socket let into the plane in which he lies, with his chin bent on the chest, and extension of the cervical vertebræ should be resorted to, immediately before the occiput is placed in the socket; the plane should also have a slight angle of inclination at first, that may be afterwards increased; for, the head being fixed, its weight does not press on the vertebræ, even when the patient lies on an inclined plane. Friction and shampooing should be employed. Leeches and blisters may be prescribed, if any pain or inflammation arise. Appropriate remedies should be adapted to the different constitutional disorders that may accompany incurvation, similar to what have been recommended in excurvation. Tonics are, in general, indicated.

"In the cases of spinal curvature in which the dorsal horizontal position is adopted, the patient should lie on an unyielding mattress, for, if placed on a yielding medium, the column would curve backwards beyond its true spinal line. To this rule there are some exceptions."

"*Lateral Curvature.*— This disease almost universally occurs *during the growth of the body*, and is almost as common as excurvation. It rarely occurs in infancy or after the adult age."

"The remote causes of lateral curvature are rachitis, or disproportion of growth of the sides of the vertebræ; unequal length of the lower extremities; undue pressure on one side of the vertebral column from carrying heavy weights on one arm, by which the spine is bent to one side; an inclined position of the body to one side long continued; muscular debility; rheumatism of the intercostal and dorsal muscles; abscess on the side of the neck or trunk; scrofula; tumors."

"In the very early stages of this disease, when the curvature is directly lateral, and there is no pain or tortuosity of the spine, a cure may be attempted, without having recourse to the recumbent posture, by proper exercises of the dorsal muscles and those of the trunk; by wearing the spine machine or collar, with lateral and axillary supports, and the rods for supporting the head, as often as the patient uses the erect attitude, and by the remedies employed for the cure of rachitis. This instrument shall be worn in all cases, whenever the patient is obliged to assume the upright posture, and if a cure cannot be effected, this instrument, varied for particular purposes, must be worn. If instruments be injurious at all, they must be so in curvatures attended with rachitis, when the bones are soft and yielding. I, therefore, revert to the subject of the use of instruments. As all the spine instruments embrace and rest upon the pelvis, as their basis of support, they have been objected to by many eminent surgeons, whilst their employment has been defended by others, and great authorities may be quoted on both sids of the question.* The bad effects are attributed to a weight or burthen being thrown on parts never intended or calculated to bear them, by which the

* No such objection applies to the spine-car of the editor, and the spine-chairs of Darwin, Shaw, and Kissam, as they do not throw the weight on the pelvis.—*Editor.*

pelvis is irreparably distorted, and females suffer, during parturition, from pelvic deformity, preventing or retarding the passage of the fœtus."

"*Angular Projection of the Spine.*—This species almost always invades the lower portion of the spinal column, and the principal seat of the malady and projection is in the lumbar vertebræ, the spinous process of the third or fourth of which generally forms the prominent angular point. I have, however, seen this form in the centre of the dorsal vertebræ. The reason why this distortion assumes the angular, instead of the curved figure, seems to be, because the lumbar vertebræ below are naturally incapable of so much flexion or of being bowed out so much as the upper vertebræ, or they offer an effectual resistance to it, from being so near a fixed point as the sacrum; hence, the vertebræ below the one disordered or deranged nearly retain their spinal line, whilst those above are removed from it.

"Since my attention has been particularly directed to this species of spinal distortion, it has occurred in my practice as frequently as excurvations or lateral curvatures.

"This distortion generally occurs in young persons from three years of age to twelve; but adults are sometimes obnoxious to it."

"*Causes of Angular Projection.*—The remote causes of this species of spinal distortion are contusions of the spine; sprains of the lumbar vertebral joints; muscular debility; pressure of the cyst of lumbar abscess; a rickety growth or malformation of the bony bridge; and, more frequently than any other, scrofulous or common inflammation of the intervertebral cartilage, and horizontal surfaces of the vertebral bodies.

"The immediate causes, being the same as of other spinal distortions, must be sought for in a structural disproportion between the anterior and posterior portions of the vertebræ or intervertebral cartilages, or in the destruction of the horizontal surfaces of one or more vertebræ, and the intervertebral cartilages situated between them."

"*Treatment.*—Many of the indications of cure enumerated for the treatment of excurvation of the spine, are, with equal propriety, applicable to the angular projection, and the mode of treatment they inculcate will be found as serviceable."

"Reposing in the facial horizontal position on a soft feather bed, will be found particularly advantageous in this variety, for, as in all the cases I have seen, except one, which involved the central dorsal vertebræ, the most projecting spinous processes were those of the lumbar or lowest dorsal vertebræ, which consequently form the seat of distortion or disease; and as, in this position, this part of the spine gravitates and inclines inwards more than in any other, so as to form even in health an artificial incurvation, by which the anterior portions of the spine are separated and the posterior more closely applied, it is easy to infer, that all weight and pressure are taken off the anterior portions of the column, by which the progressive absorption is suspended and further prevented, and that some weight of the column, by the incurvation, is transferred to the posterior portion, where it occasions such a degree of pressure, as may arrest the disproportionate growth of bone, or if not, it may conduce to its accommodation, by the tendency of its pressure to promote absorption of the parts of the vertebra above and below it, by which space will be formed to accommodate the disproportioned and enlarged vertebra, and the balance of the spinal line preserved."

This work is eminently satisfactory on all the points upon which it treats. The reader who applies to it for information concerning maladies of the spine will not be disappointed. In a small compass it embraces a large amount of practical information of which the physician must often experience the need. The republication of it in its present cheap form has placed it within the reach of every one. Dr. Mitchell's additions to it are not numerous, but in every case his notes contain valuable facts or suggestions. Y.

